# The Surgical Approach to Differentiated Thyroid Cancer

**DOI:** 10.12688/f1000research.7002.1

**Published:** 2015-11-27

**Authors:** Iain Nixon

**Affiliations:** 1 Otorhinolaryngology Head and Neck Surgery, NHS Lothian, Edinburgh, EH3 9HA, UK

**Keywords:** Differentiated thyroid cancer, Thyroid surgery, Lymph node surgery, Thyroid lobectomy, Cervical lymphadenectomy, Primary thyroid surgery

## Abstract

The incidence of thyroid cancer is increasing rapidly. A large percentage of new cases identified fall into a low-risk category. As the incidence has increased, clinical experience has confirmed that the majority of patients will have excellent outcomes and that those at risk of doing badly can be reliably identified. Treatment for thyroid cancer is predominantly surgical. The decision about how aggressively this disease should be managed has remained controversial due to the excellent outcomes irrespective of the nature of surgical procedure chosen. This article reviews the developments in our understanding of the biology of thyroid cancer and the evidence that supports the approach to management.

## Introduction

Differentiated thyroid cancer is the most common endocrine malignancy and rates are increasing worldwide ^1– 8^. Patients and clinicians are increasingly aware of the diagnosis and, with ever more cases being reported, an understanding of the most appropriate way of managing this condition is essential.

Optimal management of this disease is controversial. The reasons for the controversy include the low rates of disease-specific mortality, relatively low morbidity from therapy, and the gradual evolution in our understanding of the biology of the disease.

Low mortality rates have been a challenge for investigators with an interest in differentiated thyroid cancer. Less than 10% of patients will die of the disease within 10 years of presentation. Indeed, even recurrence rates are low, particularly in the most controversial low-risk groups. As such, randomized controlled trials of therapy have not been considered feasible ^9^. The lack of prospective trials leads clinicians to rely on retrospective data, which has inherent flaws, no matter how it is collected.

The mainstay of therapy for differentiated thyroid cancer is surgery. Although when initially attempted, thyroidectomy was associated with high rates of mortality, improvements in anaesthetic and surgical technique have resulted in thyroid surgery being extremely safe. Death following thyroid surgery is reported in <0.5% of cases ^10^. In contrast, rates of injury to the recurrent laryngeal nerve and parathyroid glands are more common (2% nerve palsy and 6% need for calcium supplements at follow up ^10^). These complications, while not life threatening, result in voice change (and occasionally tracheostomy in cases of bilateral recurrent laryngeal nerve injury) and the need for long term calcium supplementation.

The initial surgical approach to thyroid cancer was radical. Total thyroidectomy and bilateral radical neck dissection achieved macroscopic disease clearance at a cost. In particular, the cervical lymphadenectomy was associated with significant functional and cosmetic impact. The recognition that histology could predict the biological behaviour of tumors was made in the mid-20 ^th^ century. Good outcomes were described for patients with differentiated lesions of follicular cell origin (papillary carcinoma/follicular carcinoma/Hurthle cell carcinoma) in comparison with medullary or anaplastic carcinoma ^11^. This observation led to a significant change in surgical approach, with a move away from aggressive neck surgery in patients with differentiated thyroid cancer.

The reports of large institutions’ retrospective case series provided further insight into disease biology with the recognition of age, tumor size, presence of extra thyroid extension, and distant metastases as risk factors within the differentiated thyroid cancer patient group ^12– 16^. These observations provided a framework for clinicians to risk stratify their patient group into those at low, intermediate, and high risk of disease-specific death (abbreviated to AMES system of risk stratification).

Along with the interest in predicting features of patients and their tumors, which predicted the biology of disease, came scrutiny of outcomes related to management of the disease itself. Until this point, no universally agreed approach had been accepted. In 1977, a report of outcomes for 576 patients with papillary thyroid cancer (the most common differentiated thyroid cancer) recorded in the US Air Force Central Tumor Registry found that total thyroidectomy and post-operative radioactive iodine therapy were associated with lower rates of recurrence and higher survival ^17^. Clinicians would use this evidence to support an approach of total thyroidectomy and radioactive iodine for all cases of differentiated thyroid cancer. The report also found high rates of surgical morbidity following neck surgery with no impact on outcome. This finding supported an approach of primary thyroid surgery without neck dissection in those patients without evidence of regional metastases.

In contrast, other groups found that, in lower risk patients, outcomes were similar following total thyroidectomy and thyroid lobectomy ^18, 19^. The conflicting findings of the impact of the extent of initial therapy on outcome continue to this day. The surgical debate has focused on the need for aggressive primary thyroid surgery (total thyroidectomy *versus* lobectomy) ^20– 25^ and the approach to the clinically uninvolved central neck (prophylactic dissection *versus* observation) ^26– 33^.

Much research has followed since the early reports. However, prospective trials are still lacking and most recommendations are based upon retrospective data, which have been analysed by expert authors with long standing biases.

The aim of this article is to examine the contemporary approach to surgical management of differentiated thyroid cancer by analysing arguments for primary thyroid surgery and cervical lymphadenectomy.

## The aims of surgical management of differentiated thyroid cancer

The primary aim of surgical oncology is to prevent death from disease. However, for the overwhelming majority of patients with differentiated thyroid cancer, the risk of death is minimal. In addition, for many patients, occult disease will be present even after successful treatment. So the goals of therapy must be seen in the appropriate context. Risk stratification is paramount. High risk patients are treated aggressively, while low risk patients may be suitable for a less aggressive approach, and some select patients with the lowest risk disease (micropapillary carcinoma distant from the recurrent nerve or trachea) may even be candidates for an observational approach. In addition to preventing disease specific death, minimizing the chance of recurrence and preventing iatrogenic injury are key objectives for the treatment team.

## Primary thyroid surgery

The most important aim of primary surgery is to achieve complete macroscopic disease clearance and to minimize the chance that ipsilateral thyroid bed surgery will ever be required again. This requires a thyroid lobectomy as a minimum (other than for the occasional patient with isolated isthmic disease). An extra capsular thyroidectomy with preservation of the recurrent laryngeal nerve and parathyroid glands should be the standard. This will achieve disease clearance and minimize the risk of thyroid bed recurrence.

A second aim of primary surgery is to render the patient suitable for adjuvant radioactive iodine by removing all thyroid tissue. The approach to adjuvant therapy is now changing. Previously, radioactive iodine was recommended for the majority of patients with tumors of 1cm or greater. Particularly in health care systems that do not biopsy lesions smaller than 1cm, this meant that all patients were candidates for total thyroidectomy. More recently, however, the role of radioactive iodine in intermediate and low risk patients has been questioned. There is a trend away from the blanket approach of total thyroidectomy and radioactive iodine for all towards a risk-adapted, individualized approach. Although radioactive iodine is still recommended in patients with aggressive primary lesions or metastatic disease to the neck or beyond, low risk patients with small-volume primary disease and without evidence of spread have little to gain from adjuvant therapy ^34^.

Therefore, appropriately selected patients are suitable for thyroid lobectomy rather than total thyroidectomy. While preserving excellent oncological outcomes, this approach has significant potential benefits. Rates of recurrent laryngeal nerve injury, hypocalcaemia, and tracheostomy are significantly lower following such unilateral surgery.

The ideal candidate for such an approach is a young patient with uninodular disease limited to the thyroid. The risk of permanent post-operative hypocalcaemia and tracheostomy is more or less 0% following thyroid lobectomy. Recurrent nerve injury is most commonly a temporary palsy, but is permanent in around 2% of cases ^10^. Operating on one side rather than both is, unsurprisingly, associated with lower rates of morbidity ^35^. However, these patients, by definition, have a residual lobe following treatment. They are not suitable for radioactive iodine and require monitoring of the contralateral lobe in the post-operative period by ultrasound. In the long term, 5–10% of such patients will require completion thyroidectomy at some point during follow up. This is mainly due to the development of nodular disease in the residual lobe. Such disease is malignant approximately half of the time ^21^.

A number of factors make the decision of which primary procedure to offer complex. Many expert authors report extremely low complication rates following total thyroidectomy. In contrast, reports from a community setting suggest that complication rates are significantly higher for the majority of patients who are operated on outside centers of excellence ^35, 36^. Following initial therapy, the tumor marker thyroglobulin can be used during follow up to detect recurrence. This tumor marker is produced both from native thyroid tissue and persistent disease. Therefore it is less useful in patients who have had thyroid lobectomy. Due to high rates of multifocal disease within the thyroid, most authors do not recommend thyroid lobectomy if there are nodules in the contralateral lobe, even if they appear benign. This is particularly relevant in areas with a high incidence of multinodular thyroid disease and is an issue increasingly encountered due to improvements in ultrasound imaging, which now detect nodular disease in over 50% of otherwise healthy individuals ^37^.

When making a decision about primary thyroid surgery, the clinician must consider a number of factors. Risk stratification should be performed for each patient and used as a guide to selection of therapy. Tumor factors are critical, and total thyroidectomy remains the treatment of choice for those high-risk patients who will be candidates for adjuvant radioactive iodine. Surgical factors must also be considered. A patient presenting to a high-volume thyroid surgeon in a center of excellence has a significantly lower risk of suffering a complication than one who presents to a surgeon with little experience. Although an ideal solution would be the centralization of thyroid surgery to minimize the number of surgeons performing this procedure, this is not feasible for most patients. Patient factors must also be considered. Differentiated thyroid cancer commonly affects young women. This patient group is often well read, motivated, and anxious. Patients may have an idea of what they consider the treatment of choice long before they arrive in the surgical clinic.

For many patients, total thyroidectomy is the treatment of choice. When performed well, it provides excellent oncological outcomes safely. It facilitates radioactive iodine if required, allows optimal follow up using thyroglobulin as a tumor marker, and addresses concerns about multifocal disease within the gland.

Clinicians should also be aware that thyroid lobectomy offers equal oncological outcomes in appropriately selected patients. There are significant advantages, particularly when patients are managed outside high-volume surgical departments. In addition, although the use of thyroglobulin in follow up is less accurate, it can still be used effectively. It should be remembered that “low-risk” patients have a mortality rate of <5% at 20 years and recurrences are extremely rare, so the value of a highly accurate tumor marker is questionable.

**Figure 1.**
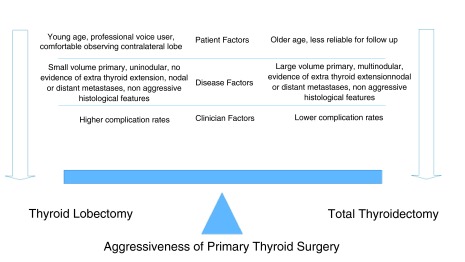
An individualized approach to selecting the aggressiveness of primary thyroid surgery.

Disease management teams should consider the issues listed above when counselling patients with differentiated thyroid cancer. By balancing tumor, clinician, and patient factors, an individualized plan can be tailored for each patient using a risk-adapted approach to optimize outcome on a case by case basis ( Figure 1).

## Lymph node surgery

Differentiated thyroid cancer and in particular papillary thyroid cancer commonly metastasizes to the neck. The most common site of metastasis is the central neck (levels VI and VII), which surrounds the thyroid gland. The second echelon of lymph nodes is the lateral neck (most commonly levels III and IV). As stated earlier, radical neck dissection was considered the treatment of choice at one point ^38^. This was an operation that was relatively quick, safe, and resulted in macroscopic disease clearance. However, it was associated with high rates of morbidity. A vogue for a much less aggressive “berry picking” approach to the removal of macroscopically involved neck nodes has largely been abandoned due to unacceptably high recurrence rates. As experience in neck surgery has improved, a compartment oriented neck dissection is recommended by most authors as the operation of choice for patients with evidence of neck disease ^39^.

Critically, the surgeon must ensure the neck has been properly evaluated prior to embarking on surgery. Ultrasound is a reliable way of assessing the lateral neck and is also the investigation of choice for the thyroid. If lateral nodal disease is encountered, imaging of the central neck may be considered using CT or MRI. Cross sectional imaging is preferable to ultrasound in assessing the central neck, particularly the mediastinal component (level VII), which is poorly visualized using ultrasound.

Those patients considered N1a or N1b (metastatic disease in the central or lateral neck respectively) following investigation should have surgery planned to remove all involved levels, and any other levels considered at risk (therapeutic neck dissection). So, those patients with disease in the central neck alone should have a central neck dissection (almost always with a total thyroidectomy as radioactive iodine is likely to be indicated).

Those patients with lateral neck involvement should have clearance of levels II-V, which are at the highest risk of metastatic involvement ^38^. Involvement of the neck above the accessory nerve and in the submental/submandibular region (level I) is uncommon, so these levels are routinely spared. In addition, differentiated thyroid cancer rarely presents with aggressive nodal disease and extra nodal extension. Therefore, in almost all patients, the sternocleidomastoid muscle, internal jugular vein, and accessory nerve can be spared. This significantly limits the morbidity of surgery and has become the standard of care for patients with lateral neck disease.

In contrast, the approach to the clinically negative neck is highly controversial. The reasons for the controversy are multiple, and again, without prospective evidence may never be resolved.

There are authors who recommend prophylactic lateral neck dissection (surgery without pre-operative evidence of involved nodes) ^28^. However, they are in the minority and the vast majority do not consider the morbidity of lateral neck surgery worth the “benefit” ^39, 40^. Despite this, if one chooses to dissect the apparently uninvolved lateral neck, metastatic disease will often be found on histology ^28^. This disease rarely manifests and if it does it can safely be salvaged at a later date. In addition, entering the lateral neck requires an extended incision and places structures at risk that are not routinely encountered in thyroid and central neck surgery (accessory nerve, marginal mandibular nerve, carotid sheath and thoracic duct).

In contrast, when performing a thyroidectomy, the central neck is, by definition, entered. The recurrent laryngeal nerves and parathyroid glands are encountered during the dissection and revision central neck surgery carries higher risks than primary procedures.

Authors who argue for prophylactic central neck surgery highlight the fact that the central neck is exposed during primary thyroid surgery. They also cite high rates of occult histopathological metastases and that such metastases “upstage” patients when identified, which gives the treating team an effective way of further risk stratifying patients to rationalize the approach to adjuvant radioactive iodine. There is some evidence that excision of the involved nodes in the central neck results in lower post-operative thyroglobulin levels, which may result in lower recurrence rates. No author has ever proven that prophylactic central neck dissection results in improved survival, as almost no patient without metastatic disease dies during follow up.

In contrast, those authors who do not support prophylactic surgery highlight the higher surgical morbidity of the procedure *versus* thyroidectomy alone, and the fact that patients who have observation rather than central neck dissection have extremely good outcomes with low rates of recurrence and extremely low rates of death. With such good outcomes enjoyed by this group of low-risk patients, the need for radioactive iodine is questionable. In addition, the approach to a central neck dissection is probably variable. Prophylactic central neck surgery involves removing tissue that lies between the recurrent laryngeal nerves. However, those centers with experience of re-operative central neck surgery find high rates of disease in areas not normally included in prophylactic surgery, such as dorsal to the recurrent laryngeal nerve or low at the thoracic inlet, which are high-risk areas for dissection and hence not included in the primary surgical field ^41^.

The controversy has resulted in ambiguity in international guidelines ^39, 40^. Such documents recommend an individualization of approach dependant on risk factors for involvement. Patients with large volume tumors and those with evidence of extra thyroid extension are at higher risk and, in such patients, guidelines recommend considering prophylactic central neck surgery without definite evidence that such an approach results in improved outcomes. In general, there is a move away from an aggressive approach where central neck dissection should be considered for all patients (with papillary thyroid cancer) and towards selecting on a case by case basis, recognizing that the degree of potential benefit cannot be calculated.

Again, in making a decision about the surgical approach to the central neck, patient, tumor, and surgeon factors must be considered. Older male patients with large volume, multicentric disease and extra thyroid extension are at higher risk of metastatic disease to the central neck, even if it is not evident on pre-operative investigation. Such patients may be considered for prophylactic central neck surgery. However, the experience of the surgeon involved should also be weighed in the decision. Morbidity for high-volume neck surgeons is lower than that for those with less experience. The potential for benefit is small, and this must be weighed against the increased risks of damage to the parathyroids and recurrent laryngeal nerves when giving advice to patients without evidence of metastatic disease in the lymph nodes (cN0).

## Conclusions

Outcomes for patients with differentiated thyroid cancer are excellent in comparison with other human cancers. Those at high risk can be easily identified from patient and tumor factors. Based upon a risk-stratified approach to managing differentiated thyroid cancer, therapeutic decisions in regard to surgery can be broken down into primary thyroidectomy and regional lymphadenectomy.

Total thyroidectomy is an operation associated with high cure rates and has been considered the gold standard internationally for years. However, thyroid lobectomy is now recognized as equally oncologically effective and is associated with lower morbidity in properly selected patients.

Compartment-oriented neck dissection has replaced both radical neck dissection and berry picking as the favoured approach to therapeutic lymphadenectomy. Outcomes for patients who have no evidence of regional metastases are now recognized as excellent, irrespective of the surgical approach. This has led to a less aggressive prophylactic surgical approach to the central neck in recent guidelines. This move away from routine prophylactic surgery has continued with the updated 2015 American Thyroid Association guidelines ^34^.

Although many treatment recommendations are now available, definitive prospective evidence to guide the thyroid surgeon is lacking in most cases. For this reason the surgical approach to this increasingly common disease remains controversial. By understanding the pros and cons of more and less aggressive approaches to the thyroid gland and the regional lymph nodes, with an understanding of the disease biology, and by applying risk stratification to all patients with differentiated thyroid cancer, appropriate decisions can be made that result in excellent oncological and surgical outcomes for the treatment team and their patients.
